# Influence of occupational exposure to pigs or chickens on human gut microbiota composition in Thailand

**DOI:** 10.1016/j.onehlt.2022.100463

**Published:** 2022-11-21

**Authors:** Duangdao Sudatip, Nadezda Mostacci, Visanu Thamlikitkul, Anne Oppliger, Markus Hilty

**Affiliations:** a Department of Occupational Health and Safety, Faculty of Public Health, Mahidol University, Bangkok, Thailand; b Institute for Infectious Diseases, University of Bern, Bern, Switzerland; c Department of Occupational and Environmental Health, Unisanté, University of Lausanne, Lausanne, Switzerland; d Faculty of Public Health, Ubon Ratchathani Rajabhat University, Ubon Ratchathani, Thailand; e Department of Medicine, Faculty of Medicine Siriraj Hospital, Mahidol University, Bangkok, Thailand

**Keywords:** Microbial communities, Pig farming, *Prevotellaceae*, One health, Allergy, Antimicrobial resistance

## Abstract

Pig farming's influence on human gut microbiota has been observed previously, but its pervasiveness is unclear. We therefore aimed at studying whether pig farming influenced human gut microbiota composition in Thailand and whether poultry farming did too.

We collected human stool samples (71 pig farmers, 131 chicken farmers, 55 non-farmers) for 16S rRNA sequencing and performed subsequent DADA2 analyses of amplicon sequence variants.

We found that Alpha diversity values were highest among chicken farmers. Relative abundances of *Prevotellaceae* were significantly higher among pig farmers than among chicken farmers and non-farmers (*p* < 0.001). Beta diversity plots revealed different clustering according to occupation. The presence or absence of antimicrobial-resistant *Escherichia coli* was not associated with changes in gut microbiota composition.

In conclusion, occupation was the strongest factor influencing gut microbiota composition in Thailand. We hypothesize that *Prevotellaceae* amplicon sequence variants are transmitted from pigs to pig farmers.

## 1 Introduction

Humans form part of a larger network comprising their immediate environment and the animals they interact with [1]. Therefore, the concept of One Health should be considered when studying the human microbiome. In Switzerland, close contact with pigs has been shown to affect pig farmers' nasal and fecal microbiota [[2], [3], [4]], highlighting the importance of considering occupation when analyzing gut microbiota composition [5]. Another important factor affecting that composition is antimicrobial use [6], as this could impact the microbiota's composition and select for antimicrobial resistant (AMR) bacteria.

The present study aimed to investigate chicken and pig farming's influence on the human gut microbiome in a northern province of Thailand. It included participants from small and very small farms who had different durations of exposure to their animals.

## 2 Material and methods

The study's overall design and sampling procedures were reported previously [7] Briefly, stool samples were collected from up to four human volunteers on small- and very small-sized pig and chicken farms (1–20 and 20–100 animals per farm, respectively). Samples from volunteers with no contact to farm animals were also included (non-farmer group). Questionnaires were administered to collect data on participants' demographic, socio-economic and health characteristics. The presence of ESC-R-*Ec* and COL-R-*Ec* in stool samples was previously investigated [8].

DNA of human volunteers' stool samples was extracted as previously described [5]. The V4 region was amplified using forward (5′-GTGCCAGCMGCCGCGGTAA-3′) and reverse (5′-GGACTACHVGGGTWTCTAAT-3′) primers modified with an Illumina adaptor sequence at the 5′ end. The resulting polymerase chain reaction products were purified and sequenced on an Illumina platform [5]. Amplicon sequencing data were analyzed using the DADA2 pipeline, as previously described. [[3], [4], [5],^9^] Briefly, amplicon sequence variants (ASVs) were calculated and taxonomies were annotated using the SILVA database (https://www.arb-silva.de/). Statistical analyses were done using ordinary one-way ANOVA for multiple comparisons, and their resulting *p*-values were adjusted (Tukey correction). Stratified analyses were performed by considering farm size (small or very small), the presence or absence of allergies among participants, and the presence or absence of ESC-R-*Ec* and COL-R-*Ec.*

For beta-diversity analyses, unweighted (Jaccard) and weighted (Ružička) distance matrices were calculated using the *vegdist* function in the *vegan* software package. Values were subsequently reduced to a two-dimensional space by non-metric multidimensional scaling (NMDS) analyses using the *metaMDS* function and visualized in R software (https://www.R-project.org/). Clustering was analyzed statistically using 1000 Monte Carlo permutation tests (PERMANOVA; the *adonis* function). Previously published microbiota data from Swiss pigs [5] were also included for additional beta-diversity analyses.

## 3 Results

### 3.1 Participant characteristics and sampling

Of 288 samples collected, 257 were finally included in the study (Table 1), consisting of 71 pig farmers, 131 chicken farmers, and 55 non-farmers. The mean ages of the participants from small farms were slightly lower than those from the very small farms, with a significant difference in the case of pig farmers (*p* < 0.01). There were participants with gastrointestinal disorders (*n* = 19) and allergies (*n* = 29). Farmers from small pig and chicken farms reported allergies significantly more often than farmers from very-small pig (Chi-squared test; X^2^ = 4.3, *p* < 0.05) and chicken farms, respectively (Chi- squared test; X^2^ = 16.4, *p* < 0.001).

**Table 1:** Study participants' characteristics.

Farmers	Farm size	Farms (n)	Samples (*n* = 257)	Median per farm (95% CI)	Mean Age (95% CI)	Female Sex	GI disorder (n = 19)⁎	Mean BMI (95% CI)⁎	Allergy (*n* = 29)⁎
Pig farmers	Very small	30	45	1.5 (1–2)	55.3 (51.9–58.7)	23 (51.1%)	6/45 (13.3%)	22.9 (21.8–24.0)	3/43 (7%)
	Small	10	26	2.5 (1–5)	46.9 (41–52.8)	11 (42.3%)	4/24 (16.7%)	23.2 (22.0–24.3)	6/24 (25%)
Chicken farmers	Very small	90	110	1 (1–2.1)	54.9 (52.3–57.5)	56 (51.0%)	4/93 (4.3%)	23.4 (22.4–23.7)	9/92 (9.8%)
	Small	10	21	2 (1–5)	47.6 (39.2–56.1)	7 (33.3%)	3/20 (15%)	23.1 (21.7–24.5)	9/19 (47.4%)
Control group	Non-Farmers		55		56.1 (52.6–59.6)	31 (56.4%)	2/50 (4%)	22.9 (22.1–23.8)	2/50 (4%)

GI = gastrointestinal, BMI = body mass index, CI = confidence interval.

⁎ Samples with missing information were excluded.

### 3.2 Differences in alpha diversity measurements and taxa

**Fig. 1:**
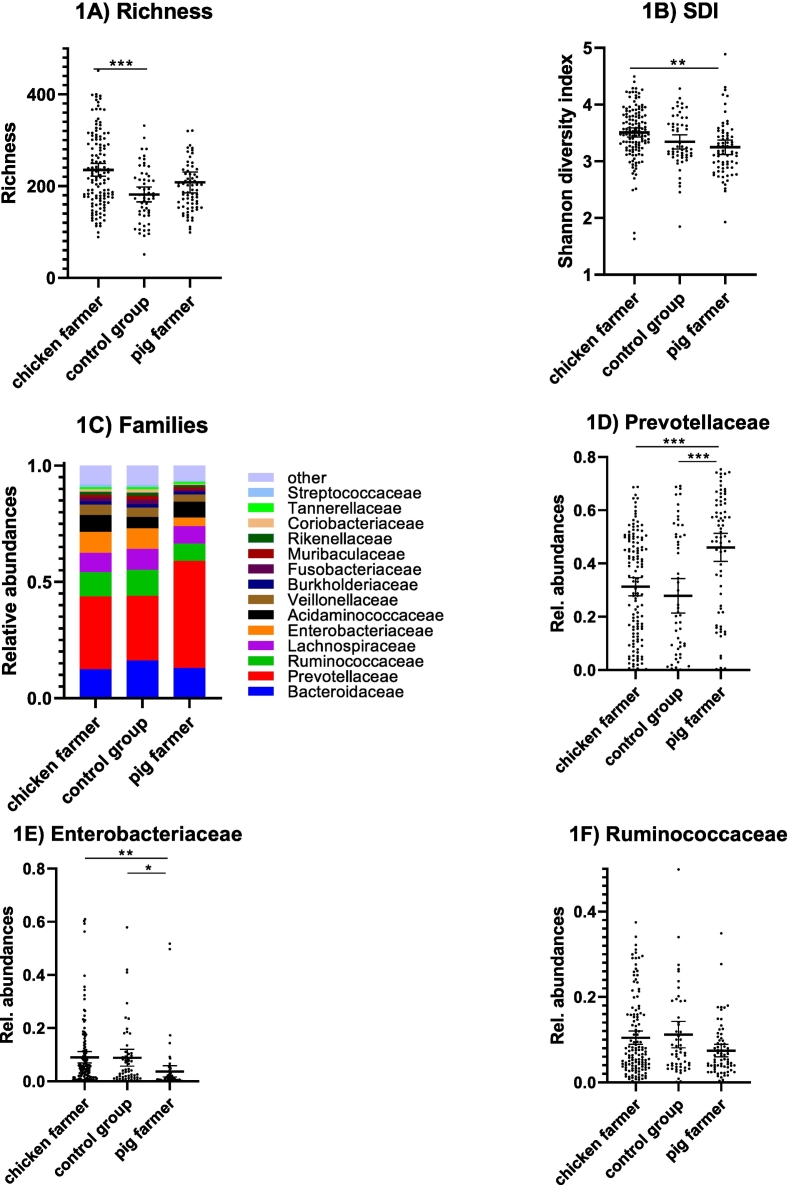
Alpha diversity and bacterial families for the three main groups. The richness (Fig. 1A) and Shannon Diversity Index (SDI) (Fig. 1B) (individual, mean, and 95% CI values) of amplicon sequence variants (ASVs) are shown for pig farmers (*n* = 71), chicken farmers (*n* = 131), and control group volunteers (*n* = 55). The relative abundances of bacterial families are shown as mean values for the three groups (Fig. 1C). Individual, mean, and 95% CI values of relative abundances of *Prevotellaceae* (Fig. 1D), *Enterobacteriacea*e (Fig. 1E), and *Ruminococcaceae* (Fig. 1F) are also plotted for a better appreciation of the differences between these three groups. Statistical analyses were done using ordinary one-way ANOVA tests for multiple comparisons. Adjusted *p*-values are indicated (*p* < 0.05 (*), *p* < 0.01 (**), and *p* < 0.001 (***)).

We found that there were significantly more ASVs among chicken farmers than among the control group (*p* < 0.001; adjusted ANOVA; Fig. 1A). Shannon Diversity Index (SDI) values were higher among chicken farmers than among pig farmers (*p* < 0.01; Fig. 1B). Taxonomic classification of ASVs based on their bacterial families revealed major shifts in composition, mainly including *Prevotellaceae*, *Enterobacteriaceae*, and *Ruminococcaceae* (Fig. 1C). Statistical analyses of *Prevotellaceae* demonstrated significantly higher relative abundances of this family among pig farmers than among chicken farmers or the control group (*p* < 0.001 and *p* < 0.001, respectively; adjusted ANOVA; Fig. 1D). In contrast, *Enterobacteriaceae* levels were lower among pig farmers than among chicken farmers (*p* < 0.01) and the control group (*p* < 0.05) (Fig. 1E). Finally, the observed differences for *Ruminococcaceae* were non-significant (Fig. 1F).

### 3.3 Beta diversity values revealed clustering according to occupation

**Fig. 2:**
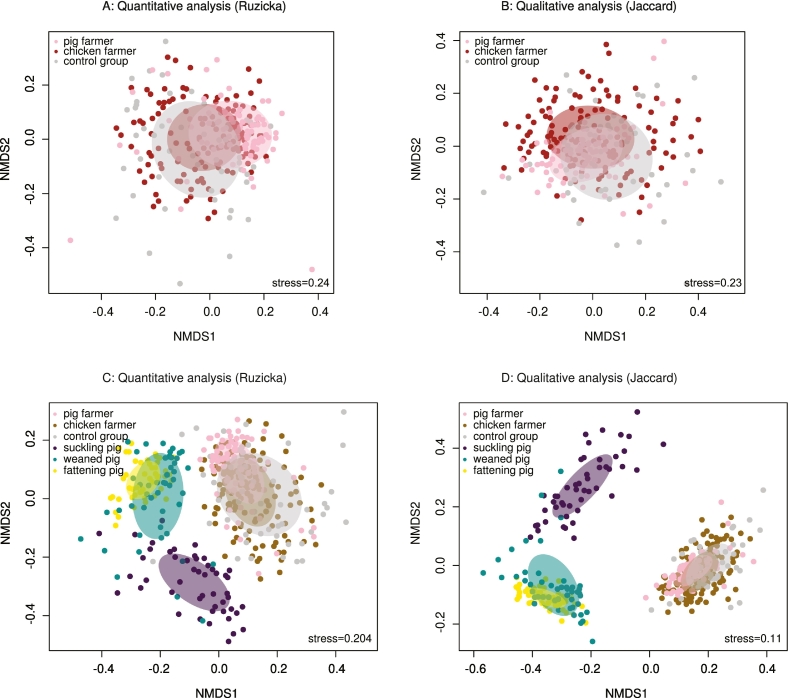
Beta diversity plots. Unweighted (Jaccard) and weighted (Ružička) distance matrices were calculated for the beta-diversity analysis of the microbiota compositions of pig farmers, chicken farmers, and control group volunteers. These values were then reduced to a two-dimensional space using non-metric multidimensional scaling (NMDS) analyses, and the outputs were visualized using R software (Fig. 2A and B). Previously published microbiota data from Swiss pigs were also included for the unweighted (Jaccard) and weighted (Ružička) analyses and then visualized (Fig. 2C and D).

We subsequently computed abundance and binary-based distance matrices to obtain non-metric multidimensional scaling plots (Figs. 2A–D). Both types of analyses revealed significant separate clusterings of samples from pig farmers and the two other groups (PERMANOVA; *p* < 0.05; Fig. 2A and B). Integrating our previous data from Swiss pigs revealed that samples from pig farmers were more similar to those of weaning and fattening pigs than were those of chicken farmers and the control group (Fig. 2C and D). This was illustrated by the 95% confidence ellipse of pig farmers being closer to these two groups of pigs than were chicken farmers and the control group (Fig. 2C and D).

### 3.4 Sub-group analyses revealed a farm-size effect

We next performed additional sub-group analyses according to farm size and found that chicken farmers' gut microbiota generally showed higher alpha diversity indices than other participant groups. Specifically, farmers on very-small chicken farms had higher richness values than the control group (*p* < 0.001; Supplementary Fig. S1A). Moreover, SDI values were lower among pig farmers on small- and very-small-sized farms than among farmers on very-small chicken farms (*p* < 0.05; Fig. S1B). Plotting microbiota compositions based on bacterial families also showed significantly more *Prevotellacecae* among pig farmers than among the other groups, except the farmers on small chicken farms (Figs. S1C and S1D). We also observed a non-significant tendency towards more *Prevotellaceae* among non-allergic individuals than among those participants reporting allergies (Fig. S1E; *p* = 0.07: unpaired *t*-test). We also investigated whether the presence of ESC-R-*Ec* or Col-R-*Ec* was associated with a change in microbiota composition. Our results showed that 44.8% (*n* = 113) and 30.6% (*n* = 77) of all our human samples were positive for ESC-R-*Ec* and COL-R-*Ec*, respectively. However, on inspecting the bacterial families, we did not observe any changes neither for the *Enterobacteriacae* family nor other families in the presence of ESC-R-*Ec* and/or Col-R-*Ec* (Fig. S1F).

## 4 Discussion

The present study showed significant differences in the gut microbiota composition of pig farmers, chicken farmers, and non-farmers. Most importantly, the relative abundance of *Prevotellaceae* was higher in the samples from pig farmers than in those of the other groups. Alpha diversity values were highest among chicken farmers.

These results confirmed earlier findings from Switzerland [5], demonstrating that the higher relative abundance of *Prevotellaceae* in pig farmers seems to be independent of other important parameters known to influence gut microbiota (e.g., diet, ethnicity and geographical environment) [[10], [11], [12]]. The two countries evidently have different farm management practices; therefore, it is remarkable that the main results were comparable.

The presence of diseases like allergies and asthma has been reported in farm workers due to the high airborne concentrations of endotoxins and bioaerosols in animal barns [13]. We found more self-reporting cases of allergies among farmers on small-sized chicken and pig farms than among very small-sized ones, probably due to greater quantities of and/or exposure to allergens and/or endotoxins. In contrast, a cross-sectional study including school-aged children from five European countries suggested inverse associations between a diagnosis of asthma and pig keeping [14]. Therefore, pig keeping could actually have a beneficial effect on human health. Notably, another study found *Prevotella* spp. more frequently in the airway microbiomes of healthy adult and child controls than in adult or child asthmatics [15]. Considering that *Prevotella* spp. are more abundant in pig farm settings, we hypothesize that certain *Prevotellaceae* ASVs might be protective for diseases like asthma. However, *Prevotella spp.* are genetically very diverse [16], so it remains to be investigated whether distinct *Prevotella spp*. (e.g., from pig farms) are more protective than others.

Interestingly, we detected no associations between participants' microbiota compositions and the presence or absence of ESC-R-*Ec* and COL-R-*Ec*. Similarly, no differences (or very few) were detected in the microbiota compositions of individuals returning from India, whether or not they were colonized by extended-spectrum cephalosporin-resistant *Enterobacteriaceae* (ESC-R-*Ent*) [17]. An individual's microbiota composition, therefore, seems to be independent of the presence of ESC-R-*Ec* and COL-R-*Ec*. Pig farmers, however, are at increased risk of ESC-R-*Ent* or COL-R-*Ent* [18]. However, it is interesting to note that the relative abundance of *Enterobacteriaceae* is actually lower in pig farmers than in other groups.

As a limitation, it needs to be noted that the study is somewhat small, in terms of sample size and the demographic analysis is also limited. This and co-analyses of other metadata should be considered in more detail in future studies.

In conclusion, significant differences in the gut microbiota composition of pig farmers as compared to chicken farmers and non-farmers were shown, highlighting the importance to consider the occupation when studying the microbiota. Moreover, according to a previous study, higher levels of *Prevotellaceae* ASVs in pig farmers than in other groups was observed. However, the consequences of these changes in human gut microbiota composition on human health need to be investigated in other studies.

## Declarartion of Competing Interest

None declared.

## Availability of data and materials

An accession number for the reads was assigned (PRJNA720940).

## Funding

A Southeast Asia–Europe Joint Funding Scheme for Research and Innovation grant (IZJFZ3–177614) was given to AO, MH and VT; a Thailand Center of Excellence for Life Sciences (TCELS) and 10.13039/501100004704National Research Council of Thailand (NRCT) grant awarded to VT and DS.

## Ethical approval

Mahidol University's Ethics Committee for the Faculty of Tropical Medicine approved the collection of biological samples and data from humans (Certification number: MUTM-2018-035-01).

## CRediT authorship contribution statement

Duangdao Sudatip: Methodology, Investigation, Writing – review & editing. Nadezda Mostacci: Data curation, Software, Methodology, Visualization, Writing – review & editing. Visanu Thamlikitkul: Project administration, Funding acquisition, Supervision, Writing – review & editing. Anne Oppliger: Project administration, Funding acquisition, Supervision, Investigation, Writing – review & editing.  Markus Hilty: Conceptualization, Methodology, Software, Project administration, Supervision, Validation, Formal analysis, Funding acquisition, Writing – original draft, Writing – review & editing.

## Data Availability

No data was used for the research described in the article.

## Appendix A Supplementary data

Supplementary figure 1: Alpha diversity and bacterial families in stratified analyses Image 1
