# Supplementary material for: Influence of occupational exposure to pigs or chickens on human gut microbiota composition in Thailand
Source: One Health. 2022 Nov 21;15:100463. doi: 10.1016/j.onehlt.2022.100463 (PMC9754954; doi:10.1016/j.onehlt.2022.100463)
Supplement: Supplementary file 1 — Supplementary figure 1: Alpha diversity and bacterial families in stratified analyses [file mmc1.docx]

**Supplementary figure: Alpha diversity and bacterial families in stratified analyses**

The richness (Figure S1A) and Shannon Diversity Index (SDI) (Figure S1B) values of amplicon sequence variants (ASVs) are shown for the stratified analyses of people from small (n = 26) and very-small (n = 45) pig farms, small (n = 21) and very-small (n = 110) chicken farms, and the control group (n = 55). The relative abundances of bacterial families are shown as the mean values for individuals from small pig farms versus those from very-small pig farms (Figure S1C).

Individual mean and 95% CI values of the relative abundances of *Prevotellaceae* are plotted for the five groups (Figure S1D). In addition, the respective bacterial families present were compared between people reporting allergies and those not (Figure S1E). Finally, participants’ bacterial families are plotted in the presence and absence of ESBL *Ec* or Colistin (Col) resistant *Ec*, respectively (Figure S1F). Statistical analyses were done using ordinary one-way ANOVA for multiple comparisons. Adjusted p-values are indicated (*p*< 0.05 (*), *p*< 0.01 (**), and *p*< 0.001 (***)
